# Intraocular Adeno-Associated Virus-Mediated Transgene Endothelin-1 Delivery to the Rat Eye Induces Functional Changes Indicative of Retinal Ischemia—A Potential Chronic Glaucoma Model

**DOI:** 10.3390/cells12151987

**Published:** 2023-08-02

**Authors:** Karin M. L. Nordahl, Vadim Fedulov, Anja Holm, Kristian A. Haanes

**Affiliations:** 1 Clinical Experimental Research, Glostrup Research Institute, Rigshospitalet, 2600 Glostrup, Denmark; anja.holm.nordvang@regionh.dk (A.H.); kristian.agmund.haanes@regionh.dk (K.A.H.); 2 Clinical and Medical Affairs, Radiometer, 2700 Brønshøj, Denmark; vadim.fedulov@radiometer.dk; 3 Center for RNA Medicine, Department of Clinical Medicine, Aalborg University, 2450 Copenhagen, Denmark

**Keywords:** AAV vector, animal model, compensatory response, electroretinography, endothelin-1, gene expression, glaucoma, retinal ischemia, transgene delivery

## Abstract

Endothelin-1 (ET-1) overactivity has been implicated as a factor contributing to glaucomatous neuropathy, and it has been utilized in animal models of retinal ischemia. The functional effects of long-term ET-1 exposure and possible compensatory mechanisms have, however, not been investigated. This was therefore the purpose of our study. ET-1 was delivered into rat eyes via a single intravitreal injection of 500 µM or via transgene delivery using an adeno-associated viral (AAV) vector. Retinal function was assessed using electroretinography (ERG) and the retinal expression of potentially compensatory genes was evaluated by means of qRT-PCR. Acute ET-1 delivery led to vasoconstriction and a significant reduction in the ERG response. AAV–ET-1 resulted in substantial transgene expression and ERG results similar to the acute ET-1 injections and comparable to other models of retinal ischemia. Compensatory changes were observed, including an increase in calcitonin gene-related peptide (CGRP) gene expression, which may both counterbalance the vasoconstrictive effects of ET-1 and provide neuroprotection. This chronic ET-1 ischemia model might be especially relevant to glaucoma research, mimicking the mild and repeated ischemic events in patients with long-term vascular dysfunction. The compensatory mechanisms, and particularly the role of vasodilatory CGRP in mitigating the retinal damage, warrant further investigation with the aim of evaluating new therapeutic strategies.

## 1. Introduction

Glaucoma, an optic neuropathic condition characterized by retinal ganglion cell (RGC) loss, optic nerve atrophy, cupping of the optic disc and visual field defects, is the leading cause of irreversible blindness in the world [1,2]. The etiology and pathogenesis of glaucomatous neuropathy are not fully understood, although many risk factors have been identified, with high intraocular pressure (IOP) having been considered the primary cause [1,3]. However, lowering the IOP does not halt the disease progression completely, and not all patients present with elevated IOP, i.e., normal tension glaucoma (NTG) [4]. Other mechanisms must therefore be at play, and multiple studies have shown a relationship between vascular dysfunction, low ocular perfusion pressure, and glaucoma pathophysiology [5,6,7,8,9,10,11]. Vascular dysfunction and, consequently, faulty autoregulation in response to perfusion pressure changes result in an instable blood supply and, as a result, mild but repeated ischemic insults. Inadequate blood flow to the retina results in varying levels of hypoxia and insufficient nutritional supply and waste removal, ultimately leading to cell death and, subsequently, progressive nerve degeneration and visual impairment [12,13]. Impaired autoregulation and hence fluctuating blood pressure in the retina could increase the sensitivity of the retina and optic nerve head to ischemic insults. This might be the primary mechanism behind NTG development and a complementary factor in high-tension glaucoma.

Elevated levels of endothelin-1 (ET-1), a very potent vasoconstrictor peptide involved in the normal autoregulatory vascular functions of the eye, have been associated with glaucomatous neuropathy [8,14,15,16,17]. The peptide appears to play an important role in vascular dysregulation, and reducing the effect of ET-1 in glaucomatous eyes can potentially have beneficial effects on ocular perfusion, preventing vasospasm and the risk of retinal ischemia [17,18]. Ischemic injuries following exogenous ET-1-induced retinal vasoconstriction have been demonstrated in animal models [19,20]. However, the vascular actions are not the only potentially damaging effects of ET-1 on the retina. The peptide may also promote the neurotoxic effects of glutamate [21,22,23], a major neurotransmitter of the retina, and could have direct neurodegenerative actions on glial and neuronal cells, reducing viability, impairing axonal transport and stimulating astrogliosis [24,25,26,27]. These non-ischemic effects likely contribute to the complex pathophysiology of glaucoma, supporting the need for therapeutic options not only aimed at lowering the IOP or, by other means, increasing perfusion. The vasodilator nitric oxide has been demonstrated to not only counteract ET-1-induced vasoconstriction but also to prevent ET-1 at an expressional level, to influence ET-1-receptor interactions and to act on the second messenger pathways, as a result also regulating the non-vasoactive effects of ET-1 [28,29]. Nevertheless, we do not have a complete view of the intrinsic compensatory and regulatory mechanism activated during ET-1 overactivity in the eye.

The vasoconstrictive effect of ET-1 and its close connection to glaucoma make it an attractive approach for developing various animal models of retinal and optic nerve ischemia. Intravitreal, perineural, and subconjunctival injections of ET-1 cause dose-dependent local vasoconstriction and can result in gliosis, demyelination, axon loss, optic disc excavation (in the rabbit), and RGC loss (in the rat), simulating glaucomatous neuropathy [20,30,31,32,33,34,35,36,37]. To simulate a chronic increase, repeated intravitreal ET-1 injections have been utilized [19,20]. This, however, results in fluctuating ET-1 levels and is coupled with an increased risk of unintentional ocular injuries and increased workload of repeated intravitreal injections. Others have used osmotic minipumps to administer ET-1 over time; however, this has been delivered retrobulbary [30,31,33]. Mi et al. looked at histological changes in the retina following long-term ET-1 exposure in a mouse model overexpressing ET-1 in endothelial cells (TET mice) [38]. This model, however, overexpresses ET-1 in the entire vasculature, not only in the retina, and since both eyes are equally affected, comparisons with an untreated contralateral eye for intra-animal control are not possible. Finally, functional evaluations are lacking in these models, both following acute and chronic ET-1 exposure. An animal model with truly chronically elevated intraocular ET-1 levels to mimic the long-term effects of retinal ischemia, and where the retinal function and endogenous responses are evaluated, could provide valuable insight into the mechanisms of glaucoma and prove useful for assessing different intervention strategies to reduce the detrimental effects of ET-1.

Therefore, we aimed to investigate retinal function over time using electroretinography (ERG) following an intravitreal ET-1 injection as an acute model of retinal ischemia. Furthermore, we aimed to develop a novel transgene ET-1 rat model with chronically and unilaterally elevated intraocular levels of ET-1. This would not only bear greater resemblance to the presumed events in glaucoma patients but also enable us to identify gradual adaptive processes that could arise in response to continuously elevated ET-1 levels. Our findings showed that transgene delivery of ET-1 produces similar ERG changes as seen following acute ET-1 injection and resemble the functional deficits seen in other models of retinal ischemia, including glaucoma. Gene expression analysis following transgene delivery indicated a compensatory response involving ET-1 receptor genes as well as the vasodilator calcitonin gene-related protein (CGRP), whose gene expression was increased in response to ET-1 overactivity.

## 2. Materials and Methods

### 2.1. Animals

Male Sprague–Dawley (SD) rats, 300 g, were used in all the studies. The animals were group-housed under standard conditions with free access to standard chow and water ad libitum. All the experiments were approved by and performed according to the guidelines of the Danish Animal Experiments Council (License: 2020-15-0201-00708).

### 2.2. In Vivo Experiments

#### 2.2.1. Acute ET-1 Injections

The purpose of the acute intraocular ET-1 injections was to evaluate the effect of intravitreally delivered exogenous ET-1 on retinal function assessed via ERG and to use this as a reference point before continuing with studying the effect of transgene ET-1 on retinal function. Twelve male SD rats were randomly divided into two groups and received an intravitreal (i.vt.) injection of either 500 µM ET-1 (Phoenix Pharmaceuticals, Karlsruhe, Germany) (*n* = 6) or vehicle PBS (*n* = 6) on day 0. The injections were delivered as 5 µL to the nasal quadrant. The right eye of each rat was injected once, and the left was used as an untreated control. The rats were then followed for 3 weeks, and the vasoactive effects and retinal function were monitored using a fundus camera and ERG measurements, respectively, on days 3 and 22. ERG was also performed at baseline, day −3, in the PBS-treated group.

In a short follow-up study, a new set of 12 male SD rats received either 500 µM ET-1 (*n* = 6) or vehicle PBS (*n* = 6) on day 0. The injections were again delivered as 5 µL to the nasal quadrant of the right eye. On day 3, the rats were euthanized and ocular fluid was collected for CGRP concentration measurements.

#### 2.2.2. Vector Selection

Before the transgene ET-1 delivery could be initiated, different vector serotypes were compared for transduction efficiency to the retina following i.vt. administration using a reporter gene. Hybrid adeno-associated virus (AAV) vectors with AAV2 serotype-based genomes and AAV5, AAV8, or AAV2 serotype-based capsids were compared. All the hybrid vectors (AAV2/5, AAV2/8, and AAV2/2) expressed enhanced green fluorescent protein (eGFP) under the control of a CAG promoter and were produced by Vector Biolabs (Malvern, PA, USA). To evaluate the efficacy of the gene delivery, the GFP expression was assessed postmortem at different time points by looking for fluorescent cells in the retinal flat mounts and cryosections. First, AAV2/5-GFP, AAV2/8-GFP, and AAV2/2-GFP were compared. A total of 14 male SD rats were divided into 3 vector groups, with *n* = 5 receiving AAV2/5-CAG-eGFP, *n* = 5 receiving AAV2/8-CAG-eGFP, and *n* = 4 receiving AAV2/2-CAG-eGFP. The AAV2/5 and AAV2/8 were delivered as either 10^10^ vg/eye (*n* = 3/serotype) or 10^9^ vg/eye (*n* = 2/serotype). The AAV2/2 was delivered as 10^10^ vg/eye. The viral vectors were administered as a single 3 or 5 µL i.vt. injection in the nasal or superior quadrant of the right eye. The rats were euthanized on week 4 or 8 following injection. Retinal flat mounts and cryosections were performed to allow for visualization of the GFP expression. Secondly, AAV2/2-GFP with and without the enhancer Woodchuck hepatitis posttranscriptional regulatory element (WPRE) were compared. WPRE was added in an attempt to increase the expression. Four male SD rats received AAV2/2-CAG-eGFP (10^10^ vg/eye) and fifteen male SD rats received AAV2/2-CAG-eGFP-WPRE (7.6 × 10^9^ vg/eye). The viral vectors were delivered as described above and the eyes were monitored regularly throughout the study using a fundus camera. The rats were euthanized on weeks 1, 2, 4, 6, and 8 following injection, and the retinal flat mounts and cryosections were evaluated for GFP expression. In addition, cryosections of the optic nerve were analyzed from 3 AAV2/2-GFP-WPRE-treated rats euthanized on weeks 4 and 8, and cryosections of the optic nerve, optic chiasma and brain were analyzed from 1 AAV2/2-GFP-WPRE-treated rat euthanized on week 8.

#### 2.2.3. Transgene ET-1 Administration

AAV2/2 with WPRE was chosen as the vector for ET-1 gene delivery to the retina to produce a constant overproduction of ET-1 in the rat eye. The mouse ET-1 gene, *mEDN1*, was chosen as the transgene to enable evaluation of the effect of transgene ET-1 expression on endogenous ET-1 production as measured via quantitative real-time polymerase chain reaction (qRT-PCR). A total of 32 male SD rats were randomly divided into one group (*n* = 24) receiving 3.2 × 10^10^ vg/eye AAV2/2-CAG-mEDN1-WPRE (AAV-ET-1) and one group (*n* = 8) receiving vehicle (PBS with 5% glycerol). Doses were delivered as a single 5 µL i.vt. injection in the nasal quadrant of the right eye. The left eye was used as an untreated control. The rats were then followed for up to 50 days, and the vasoactive effects and retinal function were monitored using a fundus camera and ERG measurements, respectively. A total of 6 rats from the AAV-ET-1-treated group and 2 rats from the vehicle group were euthanized on days 3, 8, 22, and 50. Following euthanasia, ocular fluid was collected and the retinas were frozen pending later qRT-PCR analysis.

### 2.3. Intravitreal Injections

The intravitreal injections were performed at day 0 after baseline fundus imaging using a 34G NanoFil needle and 10 µL micro syringe (World Precision Instruments, Sarasota, FL, USA). The injections were performed in fully anesthetized animals (ketamine 100 mg/kg + xylazine 5 mg/kg delivered intraperitoneally) placed on a heating pad after topical application on the cornea of a local anesthetics (oxybuprocain 0.4%, one drop). Both pupils were also treated with a mydriatic eye drop (tropicamide 1%, one drop) to enable fundus imaging prior to and after the injection. The needle was inserted through the sclera and into the vitreous behind the lens via the medial canthus (nasal quadrant) or under the upper eyelid (superior quadrant) and 3 or 5 µL was injected slowly (30–60 s) into the vitreous body. An antibiotic gel (chloramphenicol) was applied to the eye after withdrawing the needle. Finally, a lubricant (Viscotears gel, carbomer 2 mg/g, Bausch & Lomb Nordic AB, Bagsværd, Denmark) was applied to both eyes, fundus imaging was performed, and 2–5 mL of saline and 5 mg/kg carprofen were given subcutaneously (s.c.).

### 2.4. Fundus Imaging

Fundus visualization was performed and snap shots of the back of the eyes were taken on day 0 in relation to the i.vt. injections, prior to (baseline) and after the injection. Additionally, fundus imaging was performed after the ERG measurements. The Phoenix MICRON IV retinal microscope was used (Phoenix Research Labs, Pleasanton, CA, USA). For the fundus camera monitoring, the animals were anesthetized and the eyes were treated with a local anesthetic and a mydriatic eye drop, as described above. Lubricant was applied to both eyes.

### 2.5. Electroretinography

**Figure 1:**
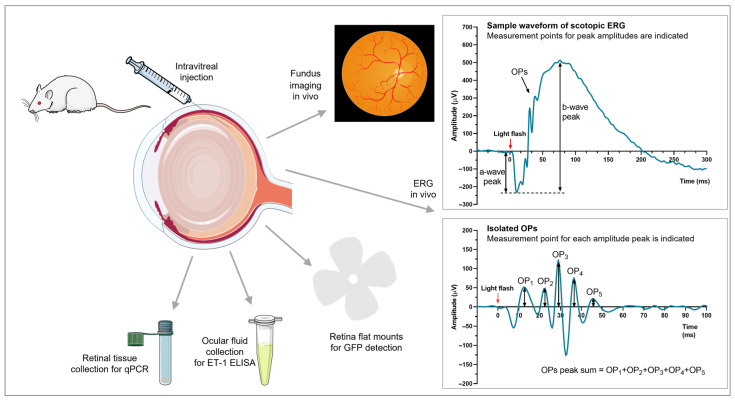
Procedures. Rat eyes were injected intravitreally with ET-1, AAV–GFP, AAV–ET-1, or vehicle. In vivo analyses consisted of fundus imaging to visualize retinal vessel diameter changes and scotopic ERG to evaluate retinal function (a-wave, b-wave, and OP peak amplitudes were calculated). Ex vivo analyses consisted of retinal flat mounts to evaluate GFP expression following transgene delivery, ocular fluid collection to measure transgene production, and qRT-PCR to assess expression of transgene and related genes. AAV, adeno-associated virus; ELISA, enzyme-linked immunosorbent assay; ERG, electroretinography; ET-1, endothelin 1; GFP, green fluorescent protein; OP, oscillatory potential; qRT-PCR, quantitative real-time polymerase chain reaction. The figure was partly generated using Servier Medical Art, provided by Servier, licensed under a Creative Commons Attribution 3.0 unported license (https://creativecommons.org/licenses/by/3.0/) accessed on 29 March 2023.

ERG analysis was performed to measure the retinal function in dark-adapted animals using the Celeris full-field rodent ERG stimulator (Diagnosys UK Ltd., Dublin, Ireland). The rats were dark-adapted overnight until the ERG analysis the next morning. Measurements were performed under general anesthesia as described above, and the eyes were treated with a local anesthetic and the pupils were dilated (same as for the fundus imaging). Lubricant was applied to both eyes and the animal was placed on a heating pad. The stimulator probes were carefully placed on the corneas of both eyes, which were covered with coupling gel, and the analysis was performed in the dark using a pre-set program. A 50/60 Hz line filter was applied to filter out electrical background noise. The program used had five flash strengths: 0.01, 0.1, 1, 3, and 5 cd·s/m^2^ of white light. The average of three measurements at each flash intensity was calculated. The generated electroretinogram was composed of an a-wave (generated by photoreceptors and postreceptoral input [39,40]), a b-wave (resulting from depolarization of post-synaptic bipolar cells and, to some degree, Müller cells [39,41,42]), and extracted oscillatory potentials (OPs) (generated/modulated by amacrine cells, bipolar cells, interplexiform cells and, possibly, RGCs [42,43]). The a-wave amplitude peak, b-wave amplitude peak (calculated from the a-wave trough), and the sum of five positive OP peaks were calculated for both eyes (Figure 1). The inter-eye difference for each parameter was calculated as the response in the right treated eye relative to the left contralateral untreated eye for each flash intensity:

$$Difference\left[\%\right]=\left((AmplitudeRIGHT/AmplitudeLEFT)\times 100\right)-100$$

The right–left difference was only calculated for the three highest flash intensities (i.e., 1, 3, and 5 cd·s/m^2^) as the amplitudes of the two initial intensities are much lower, causing a small variation to give rise to a misleadingly high percentage difference. In addition, OPs and, to some degree, a-waves were not reliably generated at the two lowest intensities. After completing the ERG program, the animal was removed from the dark room and fundus imaging was performed (except for after the pre-study baseline ERG measurements), and finally, the rat was given 2–5 mL of saline s.c. and allowed to wake up in its cage.

### 2.6. Post-Mortem Enucleation and Tissue Processing

Following euthanasia via cervical dislocation performed under full ketamine/xylazine anesthesia, the eyes were dissected out, and for one rat, also the brain, and briefly placed in PBS on ice until further processing.

#### 2.6.1. Retina Flat Mounts and Cryosections

After enucleation, the whole eye was fixed in 4% paraformaldehyde overnight at 2–8 °C. Using a dissection microscope, the eye was cut open around the limbus, the lens was removed, and four radiating cuts were made in the eye cup almost all the way down to the optic nerve, which was cut off from the backside with a deep cut to release the retina on the inside. The retina was carefully removed from the sclera, placed on a glass slide, and folded out like a four-leaf clover. Anti-fade mounting media with DAPI (Vectashield, Vector Laboratories, Newark, CA, USA) was added and cover-slipped, and the flat mount was ready for direct microscopy. After microscopy, the entire slide was place in a petri dish with PBS to allow for removal of the coverslip and release of the retina underneath. The retina was moved to 30% sucrose in PBS at 2–8 °C overnight and then frozen in Tissue-Tek O.C.T. Compound (Sakura Finetek, Torrance, CA, USA) at −80 °C. The frozen retinas were cut in 12 µm sections on a Leica cryostat at −20 °C and mounted on glass slides, dried, and stored at −80 °C. For microscopy, the slides were thawed at room temperature, carefully washed with PBS, and finally, deionized water was used to remove the O.C.T Compound and salt crystals, respectively. Anti-fade mounting media with DAPI and a cover slip were added. The GFP expression in the flat mounts and cryosections were detected using a Nikon Eclipse 80i microscope equipped with FITC and DAPI filters.

The optic nerve (once released from the retina) and brain tissue were moved from the PFA to 30% sucrose in PBS at 2–8 °C overnight, then frozen in Tissue-Tek O.C.T. Compound, and cut, mounted, and analyzed the same as the retinas.

#### 2.6.2. Ocular Fluid Collection and Retina for qRT-PCR

Ocular fluid was collected by clipping the eye open around the limbus and draining all the fluid (aqueous humor of the anterior chamber and any viscous fluid available) into a small well of a 24-well plate. Next, 20 µL of cold synthetic interstitial fluid (SIF) [44] buffer was added to the well before all the fluid was aspirated. The volume of ocular fluid collected was calculated, ocular fluid volume = total volume aspirated −20 µL SIF added, and the samples were frozen at −80 °C pending ELISA analysis. The retina was then carefully removed from the remaining eye tissue and placed in a tube with ceramic beads ready for homogenization and frozen at −80 °C pending qRT-PCR analysis. The dissections were performed on the same day as the euthanasia.

### 2.7. ELISA

The intraocular ET-1 or CGRP concentration was measured using a commercially available ET-1 ELISA kit (Enzo Life Sciences, Farmingdale, NY, USA) or a CGRP ELISA kit (Bertin Technologies, Montigny-le-Bretonneux, France), respectively, according to the manufacturer’s instructions. The ocular fluid samples were slightly diluted in SIF buffer upon freezing (see above), and for the ELISA analysis, the samples were diluted further to a final volume of 110 µL (100 µL/sample was needed). The samples were not run in duplicates due to the limited amount of sample available. The ocular fluid ET-1 or CGRP concentration was calculated using the included standard curve and considering the slightly different dilution levels of each sample.

### 2.8. Quantitative Real-Time PCR

The total RNA was extracted from the retinas using spin columns (NucleoSpin miRNA, mini kit for total RNA, MACHERY-NAGEL) in combination with QIAzol (Qiagen, Hilden, Germany) and chloroform (Sigma Aldrich, Søborg, Denmark). The samples were homogenized using QIAzol lysis buffer (Qiagen, Germany) and 1.4 mm ceramic beads (Lysing Matrix D, MP Biomedicals, Irvine, CA, USA) for 20 s at maximum speed using a FastPrep-24TM 5G instrument (MP Biomedicals, USA). The RNA concentration was measured using a Nanodrop 2000c (Thermo Fisher, Waltham, MA, USA) at 260 nm.

Next, 1 µg RNA was reversely transcribed using the iScript cDNA Synthesis Kit (Biorad, Hercules, CA, USA) according to the manufacturer’s protocol. qRT-PCR was performed using a 20× pre-designed TaqMan rat-specific gene expression assay (IDT, Coralville, IA, USA), and analyzed using the Quant-Studio 6 Pro Real-Time PCR system (Applied Biosystems, Waltham, MA, USA). The thermal cycling condition included an initial denaturation step at 50 °C for 2 min and 95 °C for 10 min, followed by 45 PCR cycles at 95 °C for 15 s and 60 °C for 1 min. The pre-designed TaqMan gene expression assay used in this study were purchased from IDT: *mmEdn1* (transgene mouse ET-1): Mm.PT.58.42871461, *rnEdn1* (endogenous rat ET-1): Rn.PT.58.44797959, *rnEdnrb* (ET_B_-receptor): Rn.PT.58.44939160, *rnEdnra* (ET_A_-receptor): Rn.PT.58.36899503, *rnCalca* (αCGRP): Rn.PT.58.38313190, *rnCalcrl* (calcitonin-receptor-like receptor): Rn.PT.58.6973608, *rnRamp1* (RAMP1, receptor activity-modifying protein 1): Rn.PT.58.36389967, *rnActb* (β-actin): Rn.PT.39a.22214838.g. The relative mRNA expression levels were normalized to β-actin and determined by calculating the 2^−ΔΔCt^.

The in vivo and ex vivo procedures are summarized in Figure 1

### 2.9. Statistical Analysis

For comparing days or groups across different light intensities, a 2-way ANOVA was used. For comparing right and left eyes within a group at different time points, with different animals at each time point, multiple paired *t*-tests were used. For comparing eyes between groups at different time points, with different animals at each time point, multiple unpaired *t*-tests were used. For the correlation analysis, Pearson correlation was performed. Differences were considered significant when *p* < 0.05. GraphPad Prism (Prism 9.3.1, GraphPad Software, La Jolla, CA, USA) was used for analyzing the data and illustrating the results.

## 3. Results

### 3.1. Acute Intravitreal ET-1 Administration

Of the six rats entering the study in the PBS-treatment group, one was euthanized prematurely due to a bad reaction to the anesthesia and was therefore excluded from the study, leaving five rats in the control group.

**Figure 2:**
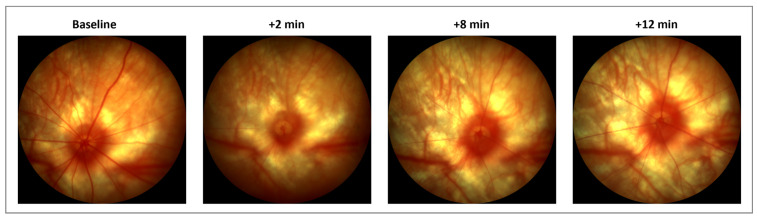
Fundus imaging of retinal vessels following an intravitreal injection of 500 µM ET-1. Pictures were taken at baseline (pre-injection) and again at 2, 8, and 12 min after the injection. Images are from a representative rat. ET-1 was delivered in the nasal quadrant, to the right in the pictures. At 2 min, complete obstruction of retinal, but not of choroidal blood vessels, is seen. At 12 min, the perfusion is clearly returning to the retinal vessels.

The acute ET-1 intravitreal injections caused an immediate and transitory constriction of the retinal vessels, as seen on the fundus imaging (Figure 2). No effect on the vessel diameter was seen after the PBS treatment. After the initial effect on the retinal blood vessels following ET-1 delivery, no changes to the fundus vessels were observed on day 3 or 22. There was also no effect on the ciliary vessel contractility in the choroid, as seen through the retina on the fundus imaging, in either group at any time point.

The ERG results showed a significant reduction in the b-wave (day 3 *p* < 0.001, day 22 *p* = 0.006) and OPs (day 3 *p* < 0.0001, day 22 *p* = 0.013), but not the a-wave, peak amplitudes for the right ET-1-treated eyes compared to the right PBS-treated eyes on both days 3 and 22 (Table 1).

**Figure 3:**
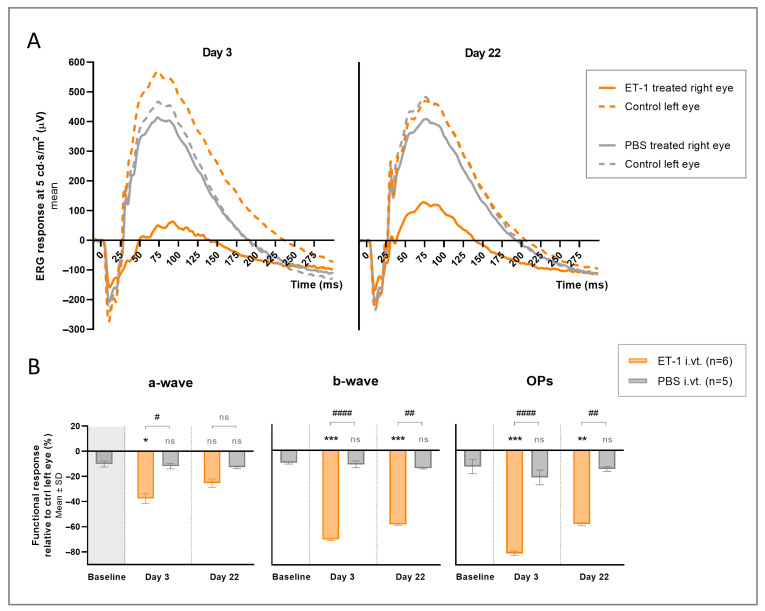
Electroretinography (ERG) results following an intravitreal injection of ET-1 or PBS. A single injection of 500 µM ET-1 (*n* = 6) or PBS (*n* = 5) was administered to the right eye on day 0, while the left eye was kept untreated. (**A**) Average ERG waveform results for right and left eyes at 5 cd·s/m^2^ are shown for both groups at days 3 and 22. (**B**) The relative differences in response in the treated right eyes compared to the contralateral untreated left eyes of each group are shown for the a-wave, b-wave, and OPs on days 3 and 22. Baseline measurements, pre-injection results, are also presented for the PBS-treated group. The mean value of the group is the mean result for all light intensities in that group (1, 3, and 5 cd·s/m^2^). Statistical comparisons are shown between the two groups (# *p* < 0.05, ## *p* < 0.01, #### *p* < 0.0001) and between each group and baseline (* *p* < 0.05, ** *p* < 0.01, *** *p* < 0.001). A 2-way ANOVA was used for comparing groups across the different light intensities. ns, nonsignificant.

The ERG waveform results for the right and left eyes at one light intensity, 5 cd·s/m^2^, are presented in Figure 3A. To eliminate daily and individual variations, the difference in the peak amplitude of the right treated eyes relative to the control fellow left eyes was calculated and compared between the two treatment groups. A significant inter-eye difference in the a-wave (*p* = 0.010), b-wave (*p* < 0.0001), and OPs (*p* < 0.0001) peak responses was seen on day 3 in the ET-1-treated group compared to the PBS-treated group (Figure 3B. Table 2). This effect was still present on day 22 but less pronounced and only reaching significance for the b-waves (*p* = 0.001), and OPs (*p* = 0.001). When comparing the ET-1-treated group to the inter-eye differences at baseline of the PBS-treated group, the difference was similar and reached significance on day 3 for the a-waves (*p* = 0.021), b-waves (*p* < 0.001) and OPs (*p* < 0.001), and on day 22 for the b-waves (*p* < 0.001) and OPs (*p* = 0.007). No significant difference was seen within the PBS-treated group when comparing the inter-eye differences at baseline to the day 3 and day 22 results.

### 3.2. Vector Selection

**Figure 4:**
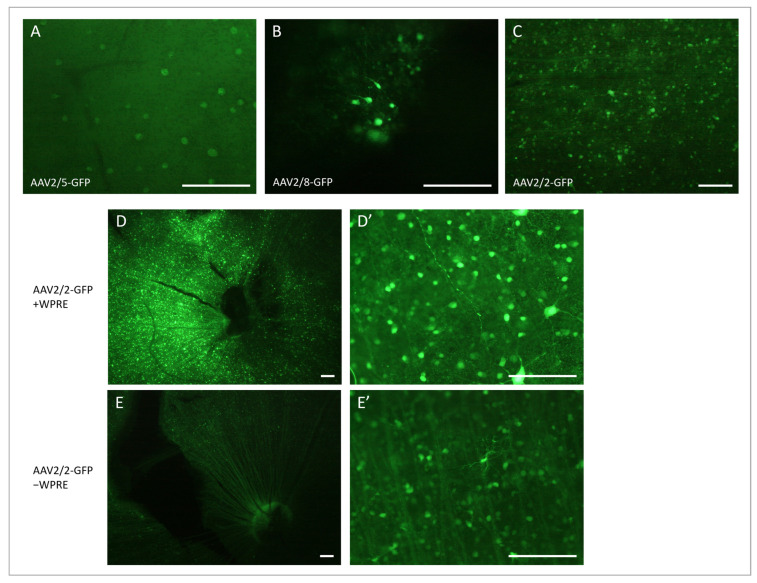
Fluorescence micrographs of retinal flat mounts after intravitreal injection of AAV vectors carrying the eGFP gene. GFP-expressing cells 4 weeks after 10^9^ vg/eye AAV2/5-CAG-eGFP in (**A**), 10^10^ vg/eye AAV2/8-CAG-eGFP in (**B**) and 10^10^ vg/eye AAV2/2-CAG-eGFP in (**C**). GFP-expressing cells 8 weeks after intravitreal injection of AAV2/2-eGFP +/− WPRE: A stronger GFP expression is seen following 7.6 × 10^9^ vg/eye AAV2/2-CAG-eGFP-WPRE (**D**,**D’**) compared to 10^10^ vg/eye AAV2/2-CAG-eGFP (**E**,**E’**). GFP-expressing cells around the papilla with the enhancer in (**D**) and without the enhancer in (**E**). Close up of GFP expressing cells and nerve fibers in the peripheral retina with the enhancer (**D’**) and without the enhancer (**E’**). WPRE, Woodchuck hepatitis posttranscriptional regulatory element. Scale bar: 100 µm.

Moving on from the acute injection of ET-1, we aimed to develop a model using AAV vectors to express ET-1 long-term. Before doing so, however, it was essential to identify the most suitable AAV vector. Following AAV2/5-GFP and AAV2/8-GFP administration, GFP expression was absent or sparse in both the retinal flat mounts and cryosections. GFP expression was only seen in the retina of four out of ten rats: one treated with AAV2/5-GFP at the lower dose and all three of the rats that had received AAV2/8-GFP at the higher dose. In all four retinas, only small areas with a few GFP-positive cells in the ganglion cell layer were identified (Figure 4A,B). In the AAV2/2-GFP-treated rats, three out of four had clear GFP expression, and in two of them, it was over a quite large area, and in one rat, GFP was observed in the entire retina. Furthermore, not only were the cell bodies fluorescent but also dendrites and axons covering long distances were observed in the AAV2/2-GFP-treated rats (Figure 4C).

**Figure 5:**
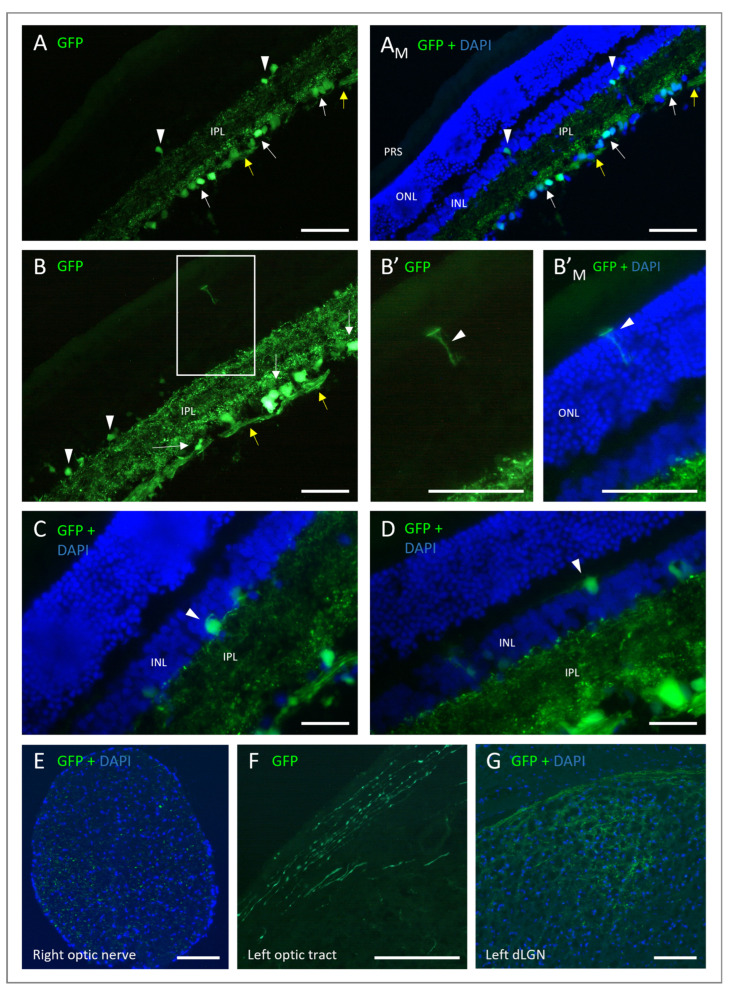
Fluorescence micrographs of retinal and brain cryosections. GFP-expressing cells at 8 weeks following injection with 7.6 × 10^9^ vg/eye AAV2/2-CAG-eGFP-WPRE. (**A**) GFP fluorescence in retina nerve fiber layer (yellow arrow), ganglion cell layer (white arrow), dendrites of the inner plexiform layer (IPL), and sporadic cells of the inner nuclear layer (INL) (white arrowhead). Merged with blue fluorescence from DAPI-stained nuclei in (**A_M_**). (**B**) Same as in (**A**). Square zoning enlarged in (**B’**). (**B’**) Insert from B with what looks like a Müller cell (white arrowhead) and merged with DAPI in (**B’_M_**). (**C**,**D**) Transduced cells of the INL (white arrowheads), GFP merged with DAPI. (**E**) Cross-section of the right optic nerve, GFP fluorescence merged with DAPI-stained nuclei. (**F**) Coronal cross-section of the brain, GFP fluorescent retinal ganglion cell fibers of the optic tract. (**G**) Coronal cross-section of the left dorsal lateral geniculate nucleus (dLGN) of the thalamus, GFP merged with DAPI. DAPI, 4′,6-diamidino-2-phenylindole; ONL, outer nuclear layer; PRS, photoreceptor segment. Scale bar: 100 µm.

Of the three vectors tested, AAV2/2 resulted in the clearest GFP expression. To further increase the expression, a WPRE enhancer was added, and the expression was compared using AAV2/2-GFP with and without WPRE. In the AAV2/2-GFP-treated rats (without enhancer), all four had clear and extensive GFP expression in 1/5 to 1/2 of the retina, as observed on the flat mounts, with GFP-positive nerve fibers gathering at the papilla and with multiple fluorescent cell bodies in the ganglion cell layer and, possibly, some inner nuclear layer cells at different depths (Figure 4E). The expression was more intense at six and eight weeks compared to four weeks after the vector injection. Following the AAV2/2-GFP-WPRE injection, 13 out of 15 rats had very clear and strong GFP expression on the retinal flat mounts. Most retinas had a very strongly fluorescent zone, with the rest of the retina sparsely dotted with some GFP-positive cells or fiber networks. Some retinas were, however, entirely filled with numerous GFP-positive cells and fibers. GFP expression was seen already on week 1 and became stronger with time, with the most intense fluorescence seen on week 8 (Figure 4D). Cryosections of the retinas confirmed the findings from the flat mounts, showing that mainly cells of the ganglion cell layer, and sporadic cells of the inner nuclear layer, had been transduced (Figure 5A–D). The GFP expression of the transduced cells could be seen throughout the entire cell, including the dendrites in the inner plexiform layer and RGC axons of the nerve fiber layer. No photoreceptors expressing GFP were observed. The GFP expression of the RGCs and their nerve fibers could be followed via the optic nerve, optic chiasma, and optic tract to the dorsal lateral geniculate nucleus (dLGN) of the thalamus (Figure 5E–G).

Adding the WPRE enhancer did have a positive effect on the transduction of the rat retina, giving rise to more intense fluorescence and a larger transfected area with more densely packed GFP positive cells compared to the AAV2/2-GFP without the enhancer.

### 3.3. ET-1 Delivery to the Rat Retina via AAV-Mediated Gene Transfer

Having determined that AAV2/2 with the WPRE enhancer was the optimal choice, we proceeded to replace the eGFP with *mEDN1* (mouse ET-1) in the vector to develop a chronically expressing ET-1 model.

**Figure 6:**
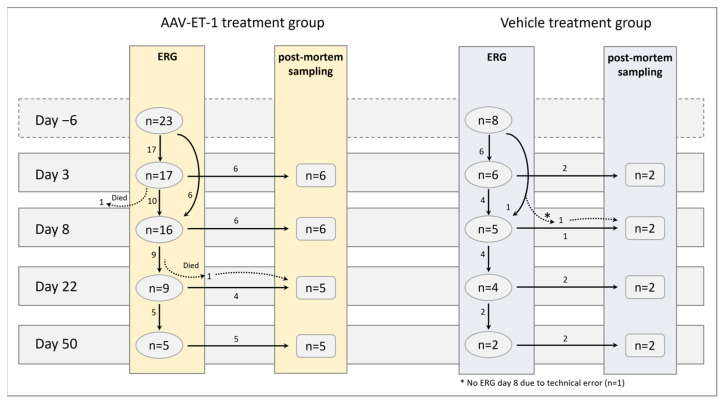
Sampling groups in the AAV-ET-1 study. Sprague–Dawley rats received an intravitreal injection of AAV-ET-1 (*n* = 23) or vehicle (*n* = 8) to the right eye on day 0. The vehicle group was limited to eight animals as the focus was on ERG and compensatory responses related to the contralateral eye. ERG measurements were performed in vivo prior to injection (baseline) day −6 and again on days 3, 8, 22, and 50. Following ERG, some of the rats were euthanized for post-mortem sample collections on days 3, 8, 22, and 50.

In the AAV-ET-1-treated group, one rat died on anesthesia day −6 (ERG baseline measurement day); thus, only 23 rats entered the study in that group. The sample sizes at the different time points for both treatment groups are illustrated in Figure 6.

**Figure 7:**
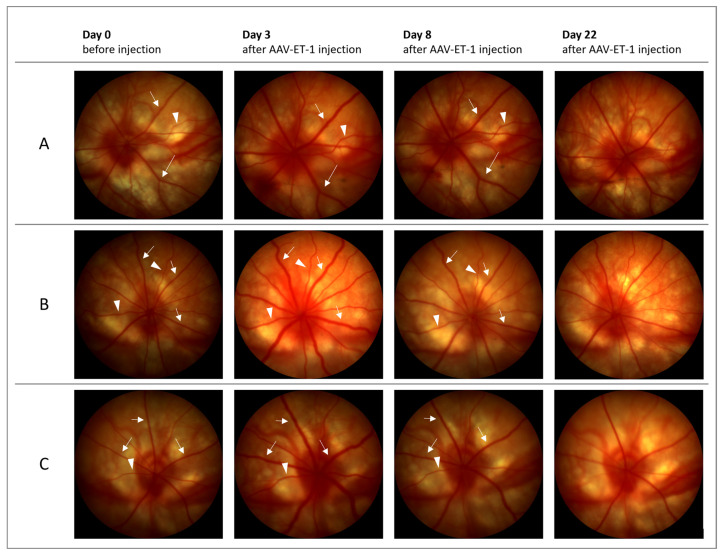
Fundus imaging before and after intravitreal AAV-ET-1 administration. Sprague–Dawley rats received an intravitreal injection of 3.2 × 10^10^ vg/eye AAV2/2-CAG-mEDN1-WPRE to the right eye on day 0. Fundus imaging of the retinal vessels was performed before injection day 0 and again on days 3, 8 and 22. Dilation of retinal veins (arrows) and arteries (arrowheads) were seen in the right injected eyes on days 3 and 8, but not on day 22, when compared to baseline. Representative images from three rats are shown (**A**–**C**). No change was seen in the contralateral untreated left eyes (not shown). *mEDN1*, mouse ET-1 gene.

Fundus imaging performed on days 3 and 8 showed retinal vessel dilation in the AAV-ET-1-treated right eyes when compared to the baseline images (16 out of 16 rats on day 3 and 13 out of 16 rats on day 8) (Figure 7, Figure S1A,B). No changes were seen in the left untreated eyes. Fundus imaging on day 22 did not show any change from baseline. On day 50, some dilation was seen in the retinal vessels of the left contralateral eyes in the AAV-ET-1-treated rats (4 out of 5 rats) (Figure S1C). No change from baseline was seen in the vehicle-treated rats on any day.

**Figure 8:**
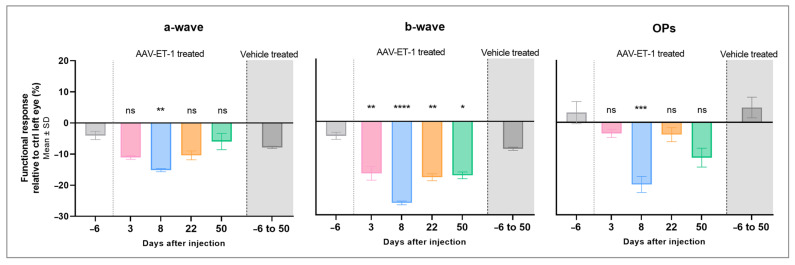
Electroretinography results following an intravitreal injection of AAV-ET-1 or vehicle. Sprague–Dawley rats received an intravitreal injection of 3.2 × 10^10^ vg/eye AAV2/2-CAG-mEDN1-WPRE (AAV-ET-1-treated) or PBS (vehicle-treated) to the right eye on day 0. ERG measurements were performed prior to injection (baseline) day −6 and again on days 3, 8, 22, and 50. The relative differences in response (peak amplitude) in the treated right eyes compared to the contralateral untreated left eyes are shown for the a-wave, b-wave, and OPs. The mean value is the average result for all three light intensities on that day (1, 3, and 5 cd·s/m^2^). Statistical comparisons for the AAV-ET-1-treated rats on each day after injection relative to the baseline results are shown. A significant reduction in all three waveforms is seen in the AAV-ET-1-treated eyes on day 8, and for the b-wave, also on days 3, 22, and 50. No significant difference was seen in the vehicle-treated group on any day when compared to the baseline measurements. These results were therefore combined in the vehicle group shown here, representing measurements on days −6, 3, 8, 22, and 50. A 2-way ANOVA was used for comparing the baseline to different days after treatment across the different light intensities. * *p* < 0.05, ** *p* < 0.01, *** *p* < 0.001, **** *p* < 0.0001; ns, nonsignificant.

The ERG results in the AAV-ET-1-treated rats showed a lower response in the right treated eyes compared to the left untreated eyes (Figure S2A–C). When comparing this inter-eye difference in peak amplitudes after treatment to differences seen at baseline, a significantly lower response in the right AAV-ET-1 treated eyes relative to the left untreated eyes was seen for the a-wave on day 8 (*p* = 0.002), for the b-wave on day 3 (*p* = 0.003), day 8 (*p* < 0.0001), day 22 (*p* = 0.001), and day 50 (*p* = 0.010), and for the OPs on day 8 (*p* < 0.001) (Figure 8, Table 3). No significant change in the ERG response was seen in the vehicle-treated rats on any day compared to baseline. Furthermore, there was no significant difference between the baseline measurements of the two groups, nor when comparing the baseline results of the AAV-ET-1 group to the combined results of all the days in the vehicle group.

**Figure 9:**
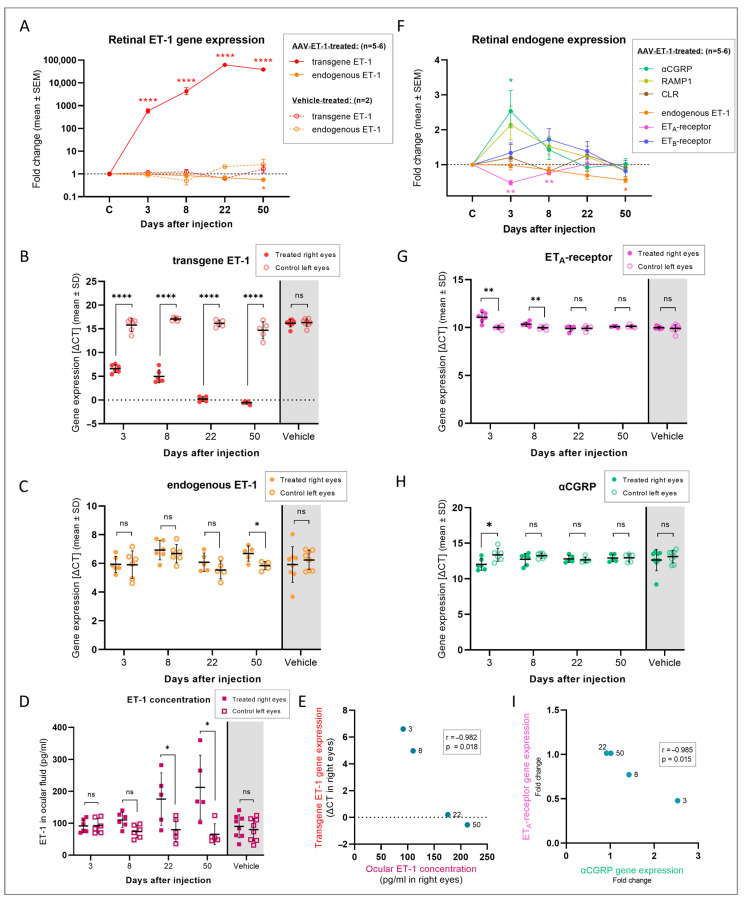
Retinal gene expression and intraocular fluid protein concentration following AAV-mediated delivery of ET-1 transgene. Sprague–Dawley rats received an intravitreal injection of 3.2 × 10^10^ vg/eye AAV2/2-CAG-mEDN1-WPRE or PBS vehicle to the right eye on day 0. Rats were euthanized on days 3, 8, 22, and 50 and ocular fluid and retinal tissue were collected. (**A**) Fold change in the transgene and endogenous *EDN1* (ET-1 gene) expression at different time points after vector or vehicle administration. For reference, the fold changes in the internal control left eyes are labeled as “C” on the timeline. Transgene (**B**) and endogenous (**C**) ET-1 gene expression in the right injected eyes compared to the left untreated eyes. Vehicle-treated rats are pooled across all four time points as no change in expression was found on any day. (**D**) ET-1 protein concentration in the ocular fluid in the right treated eyes compared to the left untreated eyes. Vehicle-treated rats are pooled across all four time points. (**E**) Correlation between the intraocular ET-1 concentration and ET-1 transgene expression in the right injected eyes over time (Pearson correlation, r = −0.982, *p* = 0.018). (**F**) Fold change in the related endogenous genes at different time points after vector administration. For reference, the fold changes in the internal control left eyes are labeled as “C” on the timeline. ET_A_-receptor (**G**) and αCGRP (**H**) gene expression in the right injected eyes compared to the left untreated eyes. Vehicle-treated rats are pooled across all four time points as no change in expression was found on any day. (**I**) Correlation between the ET_A_-receptor and αCGRP gene expression (fold change) in the retina of the AAV-ET-1-treated rats over time (Pearson correlation, r = −0.985, *p* = 0.015). Statistical comparisons in the graphs (**B**–**D**,**G**,**H**) were made using multiple paired *t*-tests, comparing the treated and untreated eyes at each time point. * *p* < 0.05, ** *p* < 0.01, **** *p* < 0.0001; ns, nonsignificant. Significant results from these comparisons are also presented in graphs (**A**) and (**F**), with the color of the asterisks and genes matching. CGRP, calcitonin gene-related protein; CLR, calcitonin-receptor-like receptor; RAMP1, receptor activity-modifying protein 1 (subunit of the CGRP receptor); ΔCT, delta cycle threshold = difference in expression between the gene of interest, i.e., transgene, and a reference gene (lower ΔCT indicates higher transgene expression).

The gene expression results revealed a massive and significant expression of transgene ET-1 in the right retina of all the AAV-ET-1-treated rats on days 3 (*p* < 0.0001), 8 (*p* < 0.00001), 22 (*p* < 0.00001), and 50 (*p* < 0.0001) following vector administration, reaching a peak in the fold change of 62,245 on day 22 (Figure 9A,B, Table S1). Transgene expression was not found in the vehicle-treated rats. Endogenous ET-1 expression was significantly lower in the AAV-ET-1-treated eyes compared to the untreated contralateral eyes on day 50 (*p* = 0.030) (Figure 9C). No change in endogenous ET-1 expression was seen in the vehicle-treated eyes. ELISA analysis of the ocular fluid from the same rats showed a significant increase in the ET-1 protein concentration of the right AAV-ET-1-treated eyes compared to their untreated left eyes on days 22 (*p* = 0.042) and 50 (*p* = 0.0027) (Figure 9D), and this correlated with the transgene ET-1 expression (Pearson correlation, r = −0.982, *p* = 0.018) (Figure 9E). No change in the intraocular ET-1 concentration was seen in the vehicle-treated rats over time.

### 3.4. Compensatory Response

To investigate the potential compensatory mechanisms, we looked at changes in the ET-1 receptor expression, and in addition, in the CGRP system due to its involvement in vasodilation and possible role in counteracting the effects of ET-1. Of the five relevant genes analyzed, the ET_A_-receptor and αCGRP genes were significantly different in the AAV-ET-1-treated right eyes compared to the fellow left eyes, whereas the ET_B_-receptor, CGRP receptor subunit RAMP1 and CGRP receptor type 1 genes were not (Figure 9F–H). No significant changes were seen in the vehicle-treated rats. ET_A_-receptor expression was significantly decreased in the right AAV-ET-1-treated eyes on day 3 (*p* = 0.003) and day 8 (*p* = 0.004) compared to their contralateral left eyes (Figure 9G). The expression of the gene for αCGRP was significantly increased on day 3 (*p* = 0.022) in the AAV-ET-1-treated right eyes when compared to the untreated left eyes (Figure 9H). Furthermore, there was a significant correlation between the ET_A_-receptor and αCGRP gene fold change variations in the AAV-ET-1-treated eyes, where αCGRP gene expression initially increased and then fell back to baseline while ET_A_-receptor gene expression displayed an initial fold change reduction which then also returned to baseline levels (Pearson correlation, r = −0.985, *p* = 0.015) (Figure 9I). There was also a significant correlation between the fold change variations of the ET_A_-receptor gene expression and *RAMP1* (Pearson correlation, r = −0.956, *p* = 0.044), and between the endogenous ET-1 gene and *RAMP1* (Pearson correlation, r = 0.986, *p* = 0.013), in the AAV-ET-1-treated group.

A final comparison was made between the untreated left eyes of the two groups, looking at each of the seven genes analyzed, and there was no significant difference in expression between the two contralateral groups for any one of the genes at any time point.

### 3.5. Follow-Up Study

**Figure 10:**
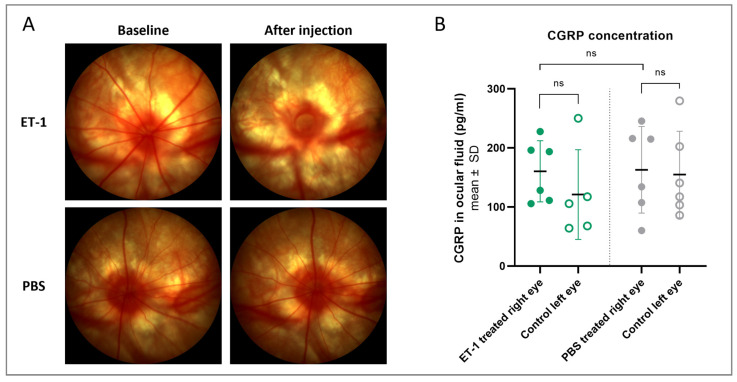
Fundus imaging and intraocular CGRP concentration following an intravitreal injection of ET-1 or PBS. A single injection of 500 µM ET-1 (n = 6) or PBS (n = 6) was administered to the right eye on day 0, while the left eye remained untreated. (**A**) Fundus imaging performed at baseline (pre-injection) and again a few minutes after the injection shows retinal vessel contraction following ET-1 injection (upper right image) but no changes following PBS treatment (lower right image). (**B**) Ocular fluid was collected on day 3 and the CGRP protein concentration was measured in ET-1 treated rats (green) and PBS-treated rats (gray). One left eye of the ET-1 treated group had to be excluded due to blood in the ocular fluid sample, confounding the assay readout. *t*-tests were used to evaluate the difference between the treated (filled circles) and untreated (open circles) eyes within groups (paired *t*-test) and between the treated eyes of the two groups (unpaired *t*-test). ns, nonsignificant (*p* > 0.05).

To investigate if CGRP compensation could play a role in the response to acute ET-1 in the eye, a short follow-up study with the injection model was performed. Again, an immediate and transitory constriction of the retinal vessels was seen following the intravitreal ET-1 injections (Figure 10A). The CGRP concentration was measured in the ocular fluid on day 3, but there was no significant difference between the PBS- or ET-1-treated eyes, nor between the treated right eyes and untreated left eyes within each group (Figure 10B). Although there was no significant effect, none of the ET-1-treated eyes had a CGRP concentration below 100 pg/mL, possibly indicating that the concentration was kept above a critical minimum level at this time point.

## 4. Discussion

The results presented in the present study show that intravitreal injections of ET-1 into the rat eye resulted in a significantly reduced scotopic flash ERG response (with lower peak amplitudes for the a-wave, b-wave, and OPs on day 3 and for the b-wave and OPs on day 22) compared to PBS treatment. Furthermore, we showed that transgene delivery of ET-1 to the rat retina was successful, with a massive and significant expression of transgene ET-1 in the retinal cells as well as an increase in the intraocular ET-1 concentration. The ERG results indicated a retinal-damaging effect similar to what was seen after the acute ET-1 injection, albeit less pronounced. A significant amplitude reduction was seen following the AAV-ET-1 treatment (with lower peak amplitudes for the a-wave on day 8, the b-wave on all four measurement days (days 3, 8, 22, and 50), and the OPs on day 8) when compared to baseline. No such changes were seen following vehicle administration.

A potential limitation of the transgene study is that the vehicle group was not injected with an empty vector to control for the potentially damaging effects of the AAV itself. However, others have shown that AAV vectors administered intravitreally do not affect the ERG in mice [45,46], nor do they cause cellular damage following subretinal injection in rats [47].

Few have looked at the ERG changes following ET-1-induced retinal damage. Ciulla et al. looked at the ERG response in the rabbit following one intravitreal injection with 1 nmol ET-1 and saw complete retinal vessel obstruction and a reduction in the a-wave amplitude, but no effect on the b-wave or OPs [48]. The same ET-1 dose administered to rabbits by Takei et al. also resulted in complete obstruction of the retinal vessels and a significant reduction in the OP amplitude, but no reduction in the a-wave or b-wave response [49]. In the present study, we observed a short-term obstruction of the retinal vessels, while the two rabbit studies had complete obstruction for roughly one hour, even though the dose used in our study was 2.5 times greater (2.5 nmol) and distributed in a much smaller intraocular volume. The explanation must lie in the morphology of the retinal vasculature. Rats have an euangiotic or holangiotic retina with a large vascular network covering the entire retina, whereas rabbits have a merangiotic vascular pattern where the retinal vessels are limited to a broad horizontal band comprising about 4% of the retina and mainly protruding into the vitreous body, not reaching particularly deep in the retinal layers [50]. The choroidal vasculature thus has a vital role in supplying almost the entire rabbit retina, whereas in the rat, it is only essential to the outer retinal structures, being gradually replaced by the retinal vessels in the central and inner retina. This discrepancy in the vascular supply might also explain the limited ERG changes seen in the two rabbit studies. This was further demonstrated in the study by Ciulla et al., where ET-1-induced ischemia was compared to ligation of the ophthalmic and ciliary arteries which caused the complete blockage of retinal and choroidal blood flow, resulting in a complete loss of the ERG response [48].

In the current study on rats, we observed a reduction in the amplitude of all three waveforms, which was more pronounced following the acute high-dose ET-1 injection compared to the chronic transgene ET-1 model. However, both models demonstrated an ERG response matching with retinal ischemia. Our results are comparable to findings in vessel occlusion models of retinal ischemia, where the a-wave and particularly the b-wave and OP amplitudes are significantly reduced [51,52,53,54]. Similarly, in human patients with unilateral central retinal vein or artery occlusion, the a-wave, b-wave and OPs are significantly reduced compared to the unaffected contralateral eye [55,56]. Many of these studies demonstrated that ERG changes precede morphological changes in the retina, illustrating the sensitivity of the ERG to retinal insults. Furthermore, all the studies indicate that the a-wave is least sensitive to retinal ischemia, which is to be expected given the different vascular supply of the outer retina. The fact that the a-wave still is affected, although to a lesser degree, could reflect the disruption of the postreceptoral portion of the a-wave [39].

Our ERG findings are also comparable to retinal ischemia models induced by raising the intraocular pressure, mimicking the effect of hypertension glaucoma, where again the a-wave, b-wave and OP amplitudes are significantly reduced [54,57,58]. Reductions in the amplitude of the OPs have been observed in patients with glaucoma [59,60], although changes to the a- and b-waves are traditionally not considered an aspect of glaucomatous damage as the inner retina with ganglion cells and the optic nerve, rather than photoreceptor and bipolar cells, are affected in glaucoma patients [61,62,63]. However, there are indications of photoreceptor cell injury in glaucoma [64,65,66], and an ischemic explanation for the outer retinal damage has been suggested [67,68]. One study found that the flash ERG measurements (i.e., a- and b-waves) were lower (smaller amplitudes and delayed peaks) in patients with glaucoma compared to optic atrophy alone, whereas the pattern ERG and OPs relating to the inner retinal function were equally affected [67]. This combination of inner and outer retinal impairment caused by vascular changes fits well with the ERG changes observed in the present study, and our results might be particularly relevant in the light of the vascular theory of glaucoma pathophysiology, suggesting that vascular abnormalities and ischemic damage play a greater role than previously expected.

There are, as mentioned in the Introduction, various non-ischemic mechanisms by which ET-1 potentially can disrupt retinal function, and these may be relevant for all models of retinal ischemia. One such possibility is via increased levels of extracellular glutamate [69,70,71,72], which ET-1 has been shown to induce by stimulating glutamate release and, possibly, also by reducing clearance by glutamate transporters [21,22,23,73,74,75]. Excess glutamate is not only neurotoxic but also effects signal transmission. Glutamate can stimulate the postsynaptic cells of the OFF pathway via non-NMDA ionotropic glutamate receptors while blocking the ON pathway via metabotropic glutamate receptors [76]. The ERG b-wave represents the activity of primarily ON-bipolar cells, and the OPs are also greatly affected by this signaling pathway [39,77]; therefore, excessive glutamate can have a negative effect on both the b-wave and OP amplitudes [78]. The reduction in the b-wave and OP peak amplitudes seen in the present study, both in the acute and the chronic models, might thus reflect the effect of ET-1 on retinal neural conductance via glutamate. That being said, the effects of ET-1 on glutamate release and uptake in the retina need more investigation, and the actions of excess glutamate on retinal cell conductance involve different cell types and multiple receptors for uptake and release, all affecting the outcome. A combination of glutamate neurotransmitter activity spurred by ET-1 and retinal damage secondary to the vascular and neurodegenerative effects of ET-1 could all be reflected in the ERG.

Following acute ET-1 injection, both the peak amplitude reductions and inter-eye differences were smaller on day 22 compared to day 3, indicating a possible recovery of retinal function with time. The ERG changes observed may thus indicate cellular dysfunction in the retina, which might recover with time when the ET-1 concentration falls back to normal, rather than cell loss [79,80,81]. This aligns with the established robustness of the retina to ischemic insults, which can be attributed to its numerous compensatory mechanisms that prevent or delay irreversible damage, such as its dual vascular supply [13,79]. In addition, in the transgene chronic model, the retinal function recovered with time and the dysfunction seemed to peak on day 8. In this case, however, the regain of function would likely not solely be due to a decreasing ET-1 concentration over time, allowing the retinal cells to recuperate, as ET-1 is continuously produced. Rather, the functional improvement could be attributed to compensatory feedback mechanisms that counterbalance and mitigate the harmful consequences of ET-1 overproduction. Consequently, we sought to examine the potential compensatory processes occurring in the retina.

We started out by looking at the regulation of the ET_A_- and ET_B_-receptors and endogenous ET-1 production. ET_A_-receptor gene expression was initially significantly decreased in the AAV-ET-1 treated eyes compared to the left untreated eyes, returning to normal by day 22. The decreased ET_A_-receptor gene expression might signify an initial response to ET-1 overproduction, serving to alleviate its contractile effect, since ET-1 primarily mediates its vasoconstrictive influence through the ET_A_-receptor on vascular smooth muscle cells [82,83]. This is supported in clinical data where a reduced level of plasma ET_A_-receptor concentration together with an increased ET-1 concentration have been observed in primary open-angle glaucoma patients; however, it was not determined which event occurred first [16]. At the same time, ET_B_-receptor binding can cause vasodilation as a negative feedback control of ET-1 vasoconstriction [84,85], and it can increase clearance of excess ET-1 [86,87]. ET_B_-receptor gene expression was also increased in the AAV-ET-1-treated eyes, returning to normal by day 50, although this change was not significant. However, the ET_B_-receptor is also associated with the neurodegenerative effects of ET-1, as ET_B_-receptor gene expression has been shown to increase in astrocytes and microglia following optic nerve injury, and ET_B_-receptor activation is thought to be involved in reactive astrogliosis [88,89]. ET_B_-receptor stimulation has also been linked to RGC death in culture [90], and ET_B_ is the main ET-1-receptor subtype of neurons and glial cells of the retina, while ET_A_ predominates in the retinal vessels [91]. Finally, expression of the endogenous ET-1 gene was significantly lower in the AAV-ET-1-treated eyes compared to the contralateral untreated eyes on the last observation day. This finding may indicate that the change in ET-1 production in response to transgene ET-1 overproduction is slow and possibly only initiated following a lengthy increase in the ET-1 concentration, unlike the receptor turnover, which is more easily regulated in response to the needs of the local environment. It is noteworthy that the other compensatory mechanisms had returned to normal levels, leaving endogenous ET-1 as the sole remaining factor of the compensation mechanism on day 50. A longer observation period, and including additional appropriate genes, might be relevant to further understand these changes.

Considering that not only reduced vasoconstriction but also vasodilation could serve as a compensatory mechanism, we shifted our focus to a vasodilating peptide, drawing from our experience with this peptide in migraine research [92]. CGRP is a powerful vasodilator [93], causing relaxation to offset the vasoconstrictive effects of ET-1 [94], and it might even also dissociate bound ET-1 via direct actions on the ET_A_-ET-1 complex, as indicated in one study [95]. Moreover, ET-1 can possibly stimulate CGRP release via ET_A_-receptor activation [73,96], thereby further promoting the compensatory role of CGRP in the ET-1 ischemic eye. Increased production of vasodilatory CGRP would therefore fit well in a counterbalancing response to the overproduction of ET-1, and indeed, our gene expression results showed an initial significant increase in αCGRP gene expression following transgene ET-1 delivery. This increase fits well with the vasodilatory response seen on fundus imaging at this time, although we have no good explanation for why CGRP gene expression then normalizes so quickly while ET-1 expression is still high. In addition, αCGRP gene expression was negatively correlated with the decrease in ET_A_-receptor gene expression. There was also a numerical increase in the expression of the CGRP receptor subunit RAMP1 gene, although this effect was not significant. Nevertheless, the trend confirms the possible involvement of the CGRP system in compensating for endothelin signaling. Finally, there are indications that αCGRP has neuroprotective actions not involving its role in hemodynamics or directly related to ET-1, and that αCGRP gene expression increases in response to neuronal injury [97,98]. Hypothesis-driven studies are needed to confirm these actions of αCGRP in ocular tissues, although CGRP and its receptors are present in the normal rat retina [99], supporting a potential protective role during ischemia and neural damage. Given the multiple routes by which CGRP can mitigate the negative effects of ET-1, we would expect the CGRP gene expression to be increased for a longer period. Perhaps its expression fluctuates and a second increase will come at a later time point.

We did not see an increase in the intraocular CGRP protein concentration on day 3 following acute ET-1 delivery. It is possible that we missed an earlier concentration surge. However, a more subtle CGRP compensatory response might still be active on day 3, possible remnants of the initial surge, but not enough to generate a significant concentration difference.

At the end of the observation period, on day 50, retinal vessel dilation was observed in the untreated eyes of the AAV-ET-1 treated rats, possibly indicating a contralateral compensatory response. Direct functional crosstalk between the left and right eye has been recorded by others, where the contralateral eye responds similarly to the treated/injured eye but without being exposed to damage or manipulation [100,101,102,103]. This can also be seen on a vascular level, and Kergoat and Lovasik demonstrated that ocular vascular stress affects the contralateral ERG [104]. We did, however, not see a significant change in the ERG responses of the contralateral eye in the AAV-ET-1 treated rats relative to baseline. Additionally, we did not see any change in gene expression over time in the contralateral eye compared to the contralateral eye of the vehicle group. A longer observation period might be needed before any contralateral effect becomes evident, particularly in SD rats with very few retino-retinal projections [102,105].

## 5. Conclusions

The present study shows that acute ET-1 injection has a clear negative impact on the rat ERG. Upon expressing ET-1 in the retina via an AAV vector, substantial quantities of ET-1 are generated, which can be detected in the ocular fluid. This induces functional impairments similar to those observed in our acute injection model and other retinal ischemia models. This novel chronic ET-1 model might be particularly relevant to glaucoma research, where similar functional changes are observed and increased ET-1 activity presumably plays an important role. Increasing the ET-1 release chronically may also better mimic the mild and repeated ischemic events in patients, where long-term vascular inadequacy with time may cause retinal damage. However, to validate this as a true glaucoma model, the pathology also needs to match, as the RGC loss and optic nerve atrophy are the hallmarks of glaucoma diagnostics and morphological assessment was not included in the present study. The changes we see relating to ET-1 overactivity can, however, still portray one aspect of the etiology behind glaucomatous ischemia. With regard to the vasodilatory response seen on the early days in the chronic model, we do not see this as incompatible with a model of retinal ischemia, as we expect compensatory mechanisms to respond to increased ET-1 levels.

Finally, leading into the potential mitigation of the retinal damage, we also show indications of compensatory changes following ET-1 overexpression. In this study, we find the alterations in CGRP gene expression particularly intriguing, and they warrant further investigation in this model with the aim of evaluating novel therapeutic interventions for retinal ischemia.

## Figures and Tables

**Table 1:** ERG peak amplitudes following an intravitreal injection of 500 µM ET-1 or PBS to the right eye of Sprague–Dawley rats.

Peak Amplitudes. Right Eye (Mean µV ± SD)						
Wave- form Light Intensity *	Day 3			Day 22		
	ET-1-Treated	PBS-Treated	*p*-Value ^#^	ET-1-Treated	PBS-Treated	*p*-Value ^#^
a-wave			ns			ns
0.1	−101.7 ± 28.7	−108.2 ± 25.3		−102.7 ± 36.7	−94.5 ± 22.9	
1	−139.1 ± 37.1	−166.6 ± 37.3		−147.7 ± 42.0	−166.4 ± 36.2	
3	−146.7 ± 29.7	−190.3 ± 35.7		−158.0 ± 52.0	−189.0 ± 46.6	
5	−159.7 ± 39.8	−207.9 ± 46.2		−166.9 ± 54.1	−205.4 ± 49.8	
b-wave			<0.001			0.006
0.1	152.8 ± 70.7	489.0 ± 153.1		241.1 ± 124.8	479.2 ± 114.0	
1	201.2 ± 68.1	574.6 ± 153.6		286.0 ± 129.2	568.7 ± 128.7	
3	217.8 ± 63.0	589.7 ± 158.4		294.4 ± 127.0	595.9 ± 141.0	
5	228.2 ± 79.5	624.5 ± 162.1		299.1 ± 136.3	619.3 ± 147.2	
OPs			<0.0001			0.013
1	40.0 ± 8.9	157.0 ± 47.8		117.2 ± 74.2	199.1 ± 33.7	
3	55.6 ± 9.9	217.0 ± 58.7		131.8 ± 67.1	263.2 ± 54.9	
5	61.1 ± 11.1	207.4 ± 49.9		137.6 ± 75.7	264.0 ± 44.6	

*, light intensities: 0.1, 1, 3, and 5 cd·s/m^2^. ^#^, 2-way ANOVA comparing groups across all light intensities. ERG, electroretinography; ET-1, endothelin-1; ns, nonsignificant; OPs, oscillatory potentials; SD, standard deviation.

**Table 2:** Relative ERG peak amplitude results in the right eyes, following an intravitreal injection of 500 µM ET-1 or PBS, compared to the untreated left eyes of Sprague–Dawley rats.

Relative Functional Change. Inter-Eye Difference (Mean% ± SD) *							
Waveform	Baseline	Day 3			Day 22		
	PBS-Treated	ET-1-Treated (*p*-Value) ^#^	PBS-Treated (*p*-Value) ^#^	*p*-Value ^†^	ET-1-Treated (*p*-Value) ^#^	PBS-Treated (*p*-Value) ^#^	*p*-Value ^†^
a-wave	−10.2 ± 2.3	−37.6 ± 3.9 (0.021)	−11.8 ± 2.2 (ns)	0.010	−25.4 ± 3.3 (ns)	−12.8 ± 1.1 (ns)	ns
b-wave	−9.9 ± 1.0	−70.1 ± 0.7 (<0.001)	−11.1 ± 2.7 (ns)	<0.0001	−58.3 ± 0.6 (<0.001)	−14.1 ± 0.5 (ns)	0.001
OPs	−12.8 ± 5.6	−81.0 ± 1.7 (<0.001)	−21.4 ± 5.8 (ns)	<0.0001	−58.0 ± 1.0 (0.007)	−14.7 ± 1.9 (ns)	0.001

*, $Difference\left[\%\right]=\left((AmplitudeRIGHT/AmplitudeLEFT)\times 100\right)-100$. Mean ± SD was calculated across three light intensities (1, 3, and 5 cd·s/m^2^). ^#^, a 2-way ANOVA comparing baseline to ET-1-treated and to PBS-treated on day 3 and day 22 across the three light intensities. ^†^, a 2-way ANOVA comparing ET-1-treated to PBS-treated on days 3 and 22 across the three light intensities.

**Table 3:** Relative ERG peak amplitude results in the right eyes, following an intravitreal injection of AAV-ET-1 or vehicle, compared to the untreated left eyes of Sprague–Dawley rats.

Relative Functional Change. Inter-Eye Difference (Mean% ± SD) *						
Waveform	AAV-ET-1 Treatment					Vehicle Treatment
	Day −6 Baseline	Day 3 (*p*-Value) ^#^	Day 8 (*p*-Value) ^#^	Day 22 (*p*-Value) ^#^	Day 50 (*p*-Value) ^#^	All Days Combined (*p*-Value) ^†^
a-wave	−4.0 ± 1.3	−11.1 ± 0.6 (ns)	−15.2 ± 0.5 (0.002)	−10.4 ± 1.4 (ns)	6.0 ± 2.6 (ns)	−7.9 ± 0.4 (ns)
b-wave	−4.6 ± 1.2	−16.7 ± 2.2 (0.003)	−26.2 ± 0.6 (<0.0001)	−17.9 ± 1.1 (0.001)	−17.3 ± 1.1 (0.010)	−8.8 ± 0.5 (ns)
OPs	3.1 ± 3.6	−3.6 ± 1.3 (ns)	−20.2 ± 2.6 (<0.001)	−4.0 ± 2.3 (ns)	−11.5 ± 3.1 (ns)	4.8 ± 3.3 (ns)

*, $Difference\left[\%\right]=\left((AmplitudeRIGHT/AmplitudeLEFT)\times 100\right)-100$. Mean ± SD is calculated across three light intensities (1, 3, and 5 cd·s/m^2^). ^#^, a 2-way ANOVA comparing each day to baseline measurements across the three light intensities. ^†^, a 2-way ANOVA comparing vehicle-treated at all time points combined to AAV-ET-1-treated at baseline across the three light intensities.

## Data Availability

Data will be made available on request.

## Supplementary Materials

The following supporting information can be downloaded at https://www.mdpi.com/article/10.3390/cells12151987/s1, Supplemental Figure S1A–C: Fundus images taken prior to and after intravitreal delivery of transgene ET-1, Figure S2A–C: Individual ERG results for the peak amplitudes in the transgene and vehicle-treated eyes relative to their contralateral untreated eyes, Table S1: RT-qPCR results for the transgene ET-1 gene expression.
