# Supplementary material for: Intraocular Adeno-Associated Virus-Mediated Transgene Endothelin-1 Delivery to the Rat Eye Induces Functional Changes Indicative of Retinal Ischemia—A Potential Chronic Glaucoma Model
Source: Cells. 2023 Aug 2;12(15):1987. doi: 10.3390/cells12151987 (PMC10417058; doi:10.3390/cells12151987)
Supplement: Supplementary file 1 [file cells-12-01987-s001.zip › Supplemental Figures - Proof (01.08.2023) - submitted.pdf]

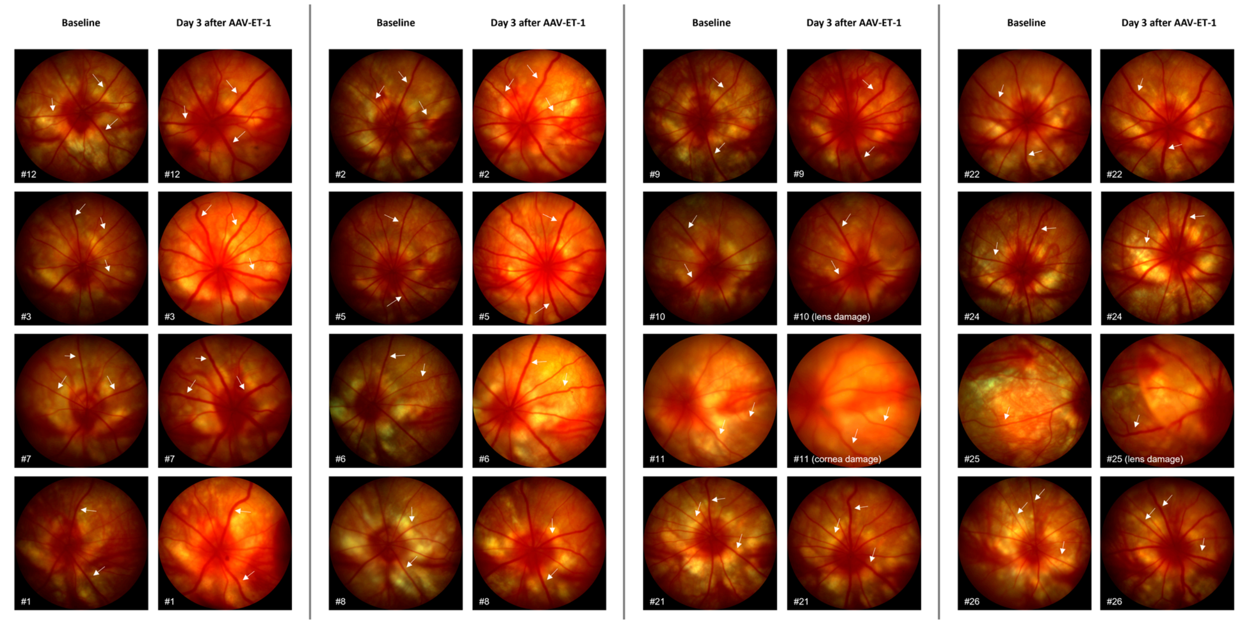

Supplemental Figure S1A. Fundus images taken at baseline (day 0) and on day 3 following intravitreal delivery of transgene ET-1 (AAV2/2-CAG-mEDN1-WPRE) to Sprague Dawley rats. Arrows indicate vessels dilated day 3. Dilation was seen in 16 out of 16 rats. AAV; adeno-associated virus, ET-1; endothelin 1

Figure S1A. Fundus images taken prior to and on day 3 after intravitreal delivery of transgene ET-1.

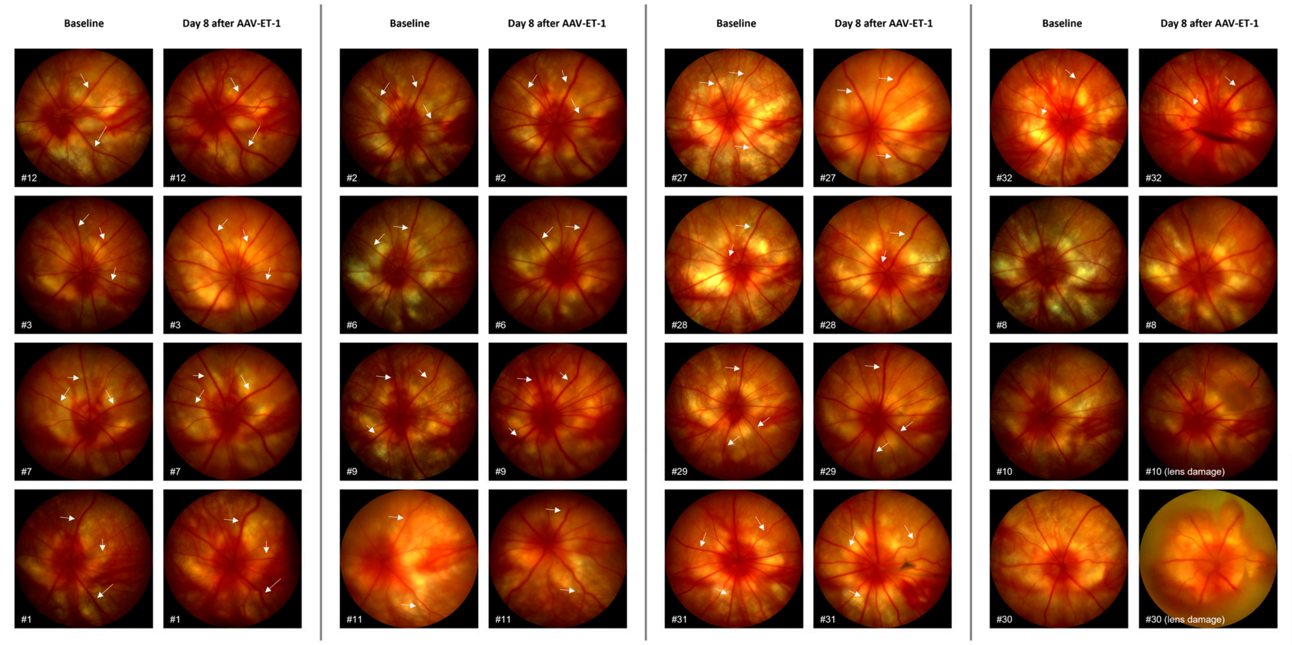

Supplemental Figure S1B. Fundus images taken at baseline (day 0) and on day 8 following intravitreal delivery of transgene ET-1 (AAV2/2-CAG-mEDN1-WPRE) to Sprague Dawley rats. Arrows indicate vessels dilated day 8. Dilation was seen in 13 out of 16 rats.

Figure S1B. Fundus images taken prior to and on day 8 after intravitreal delivery of transgene ET-1.

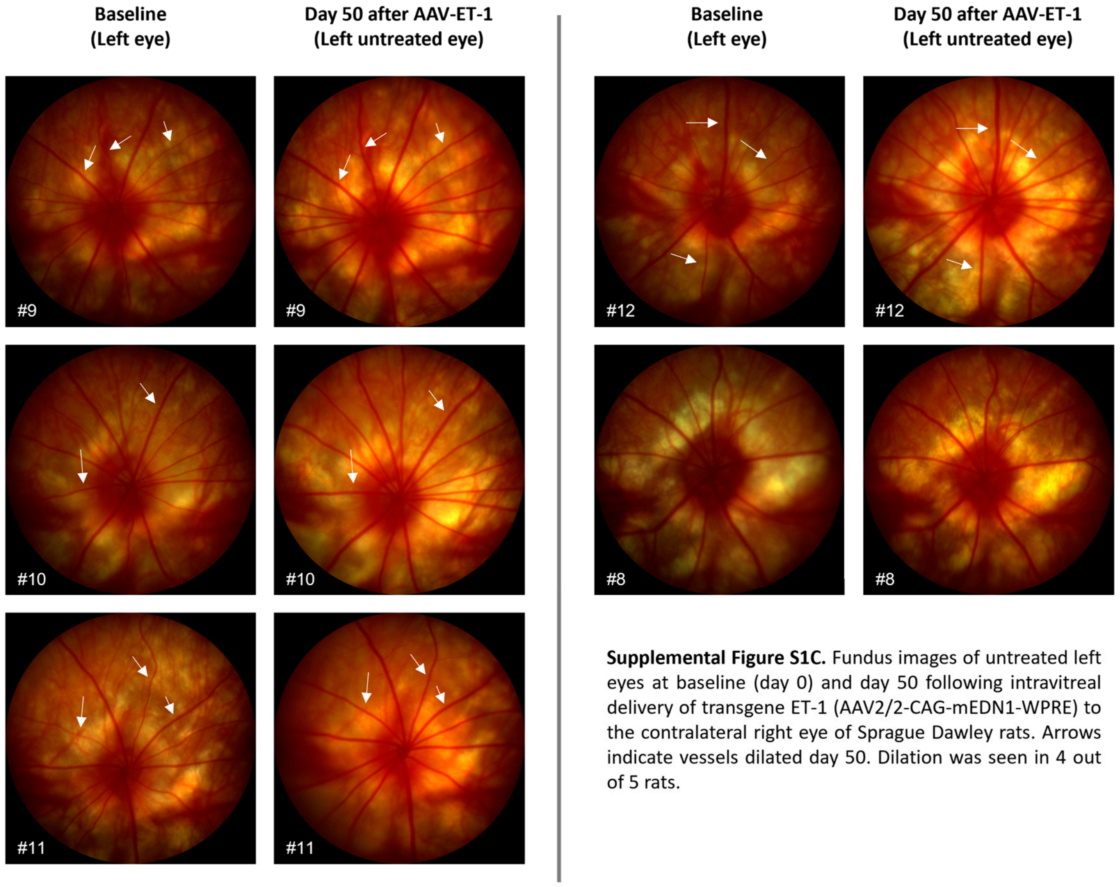

Supplemental Figure S1C. Fundus images of untreated left eyes at baseline (day 0) and day 50 following intravitreal delivery of transgene ET-1 (AAV2/2-CAG-mEDN1-WPRE) to the contralateral right eye of Sprague Dawley rats. Arrows indicate vessels dilated day 50. Dilation was seen in 4 out of 5 rats.

Figure S1C. Fundus images taken prior to and on day 50 after intravitreal delivery of transgene ET-1 to the contralateral eye.

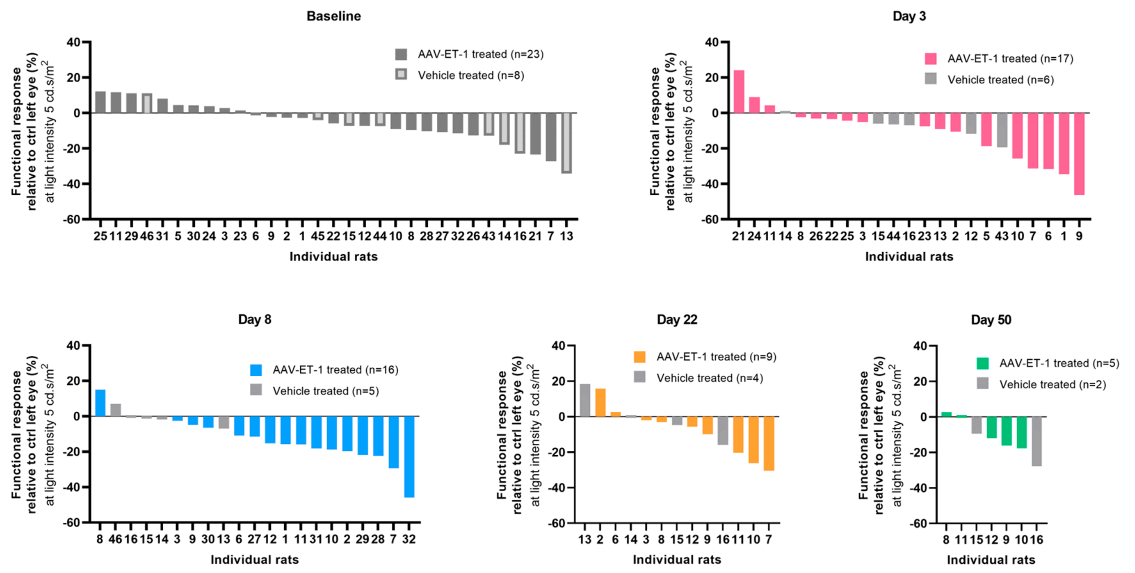

Supplemental Figure S2A. Individual electroretinography (ERG) results following transgene delivery of ET-1 to the rat retina. Sprague Dawley rats received an intravitreal injection of $3.2x10^{10}$vg/eye AAV2/2-CAG-mEDN1-WPRE (AAV-ET-1-treated) or PBS (vehicle-treated) to the right eye on day 0. ERG measurements were performed prior to injection (baseline) day -6 and again on days 3, 8, 22, and 50. The relative difference in a-wave response (peak amplitude) in treated right eyes compared to contralateral untreated left eyes are shown at the highest light intensity used (5 cd.s/m^2). AAV; adeno-associated virus, ET-1; endothelin 1

Figure S2A. Individual ERG results for a-wave peak amplitudes in transgene and vehicle-treated eyes relative to their contralateral untreated eyes.

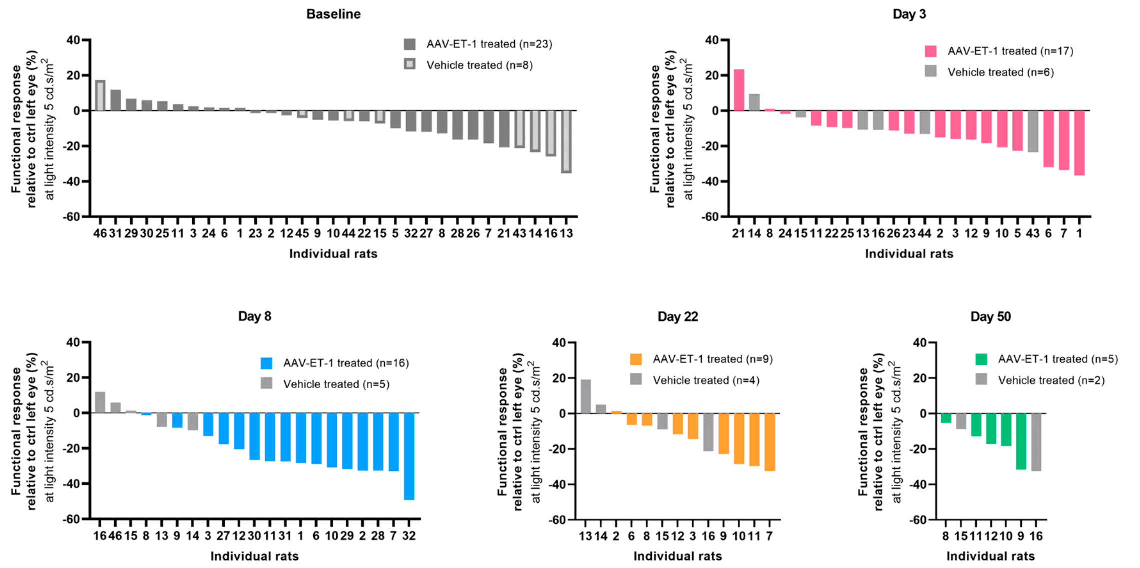

Supplemental Figure S2B. Individual electroretinography (ERG) results following transgene delivery of ET-1 to the rat retina. Sprague Dawley rats received an intravitreal injection of 3.2×10^{10}vg/eye AAV2/2-CAG-mEDN1-WPRE (AAV-ET-1-treated) or PBS (vehicle-treated) to the right eye on day 0. ERG measurements were performed prior to injection (baseline) day -6 and again on days 3, 8, 22, and 50. The relative difference in b-wave response (peak amplitude) in treated right eyes compared to contralateral untreated left eyes are shown at the highest light intensity used (5 cd.s/m²).

Figure S2B. Individual ERG results for b-wave peak amplitudes in transgene and vehicle-treated eyes relative to their contralateral untreated eyes.

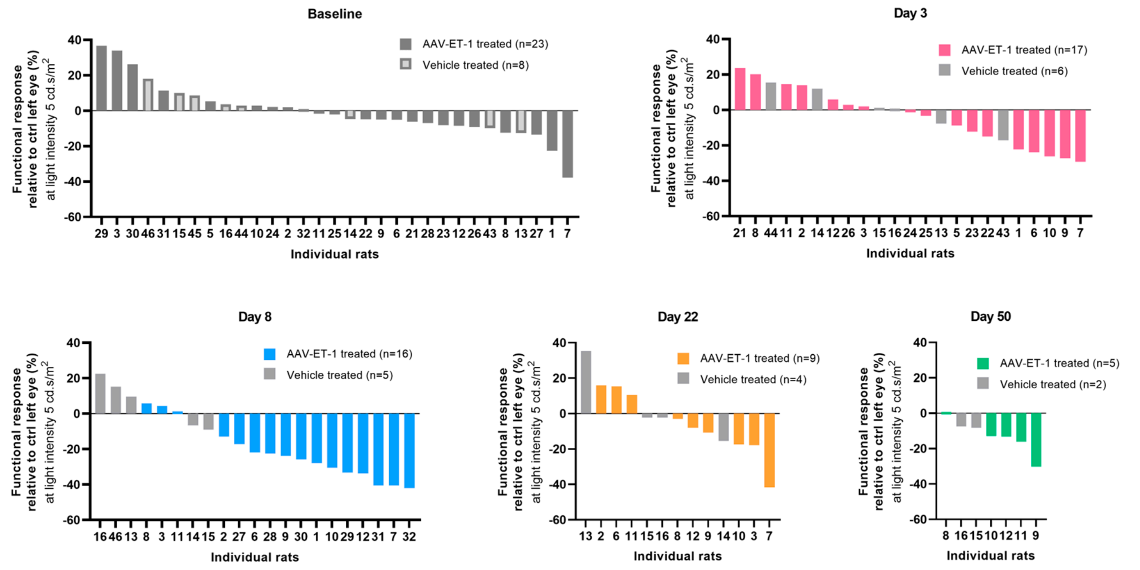

Supplemental Figure S2C. Individual electroretinography (ERG) results following transgene delivery of ET-1 to the rat retina. Sprague Dawley rats received an intravitreal injection of 3.2×10^{10}vg/eye AAV2/2-CAG-mEDN1-WPRE (AAV-ET-1-treated) or PBS (vehicle-treated) to the right eye on day 0. ERG measurements were performed prior to injection (baseline) day -6 and again on days 3, 8, 22, and 50. The relative difference in OPs response (peak amplitude) in treated right eyes compared to contralateral untreated left eyes are shown at the highest light intensity used (5 cd.s/m²).

Figure S2C. Individual ERG results for OPs peak amplitudes in transgene and vehicle-treated eyes relative to their contralateral untreated eyes.
