# Supplementary material for: Intraocular Adeno-Associated Virus-Mediated Transgene Endothelin-1 Delivery to the Rat Eye Induces Functional Changes Indicative of Retinal Ischemia—A Potential Chronic Glaucoma Model
Source: Cells. 2023 Aug 2;12(15):1987. doi: 10.3390/cells12151987 (PMC10417058; doi:10.3390/cells12151987)
Supplement: Supplementary file 1 [file cells-12-01987-s001.zip › Supplemental Table S1 - RT-qPCR results - Proof.pdf]

Supplemental Table S1. RT-qPCR results for transgene ET-1 gene expression.

Sprague Dawley rats recieved 3.2x10^{10} vg/eye AAV2/2-CAG-mEDN1-WPRE (AAV-ET-1, n=22) or vehicle (n=8) on day 0, delivered intravitreally to the right eye. Contralateral eyes were left untreated. Transgene mouse ET-1 gene (mEDN1) expression was measured using RT-qPCR on retinal tissue from right (DEX) treated and left (SIN) untreated eyes collected on different days after the injection.

			mean CT		mean CT		ΔCT		ΔΔCT		Fold change	
			Transgene (mouse ET-1)		Reference gene (B-actin)		Normalized Transgene (mouse ET-1)					
	Sample#	Euthanazia timepoint:	DEX	SIN	DEX	SIN	DEX	SIN	DEX	SIN	DEX	SIN
AAV-ET-1	1	Day 3	22.79	34.05	16.56	17.55	6.22	16.50				
	2		23.42	33.85	15.82	16.87	7.60	16.99				
	3		24.24	31.01	16.95	17.48	7.29	13.53				
	4		22.69	32.03	16.69	17.23	6.00	14.80				
	5		22.76	33.18	15.74	16.90	7.01	16.28				
	6		21.55	34.38	16.12	17.73	5.44	16.65				
		Average:	22.91	33.08	16.31	17.29	6.59	15.79	-9.20	0.00	586.72	1.00
	7	Day 8	20.92	34.88	16.84	17.59	4.08	17.29				
	8		21.07	34.21	16.98	17.60	4.09	16.61				
	9		21.27	35.08	17.42	17.67	3.85	17.41				
	10		21.74	34.73	17.03	17.56	4.71	17.17				
	11		24.31	33.66	16.98	16.98	7.33	16.68				
	12		22.55	34.68	16.73	17.51	5.82	17.17				
		Average:	21.98	34.54	17.00	17.49	4.98	17.05	-12.07	0.00	4,309	1.00
	13	Day 22	17.15	33.65	17.54	17.56	-0.39	16.08				
	14		17.44	34.26	17.67	17.57	-0.23	16.70				
	15		18.32	34.25	17.60	17.28	0.73	16.97				
	16		17.49	33.32	17.32	17.67	0.16	15.65				
	17		18.67	33.28	17.93	18.04	0.74	15.24				
		Average:	17.81	33.75	17.61	17.63	0.20	16.13	-15.93	0.00	62,245	1.00
	18	Day 50	16.56	34.30	17.18	17.49	-0.62	16.81				
	19		16.32	32.20	17.41	17.15	-1.08	15.05				
	20		16.62	28.64	16.94	16.56	-0.32	12.08				
	21		17.03	33.18	17.27	17.55	-0.23	15.64				
	22		16.68	31.40	17.22	17.55	-0.54	13.86				
		Average:	16.64	31.95	17.20	17.26	-0.56	14.69	-15.25	0.00	38,865	1.00
Vehicle	23	Day 3	33.17	34.11	16.83	16.99	16.34	17.12				
	24		34.12	33.72	17.17	17.09	16.95	16.63				
		Average:	33.64	33.92	17.00	17.04	16.65	16.87	-0.23	0.00	1.17	1.00
	25	Day 8	33.35	34.00	16.42	16.82	16.92	17.18				
	26		32.84	32.42	16.88	16.10	15.96	16.32				
		Average:	33.09	33.21	16.65	16.46	16.44	16.75	-0.31	0.00	1.24	1.00
	27	Day 22	34.36	32.39	17.84	17.69	16.52	14.70				
	28		33.76	34.30	17.84	17.85	15.92	16.45				
		Average:	34.06	33.34	17.84	17.77	16.22	15.57	0.65	0.00	0.64	1.00
	29	Day 50	33.96	33.86	17.81	17.71	16.15	16.15				
	30		32.02	32.90	17.53	16.97	14.49	15.93				
		Average:	32.99	33.38	17.67	17.34	15.32	16.04	-0.72	0.00	1.65	1.00

mean CT = Mean cycle threshold of three repetetive measurements

ΔCT (delta cycle threshold) = difference in expression between gene of interest, i.e. transgene, and a reference gene (B-actin)

ΔΔCT = difference between ΔCT of treated right eye and untreated control left eye. This value is used to calculate fold change

Fold change = Ratio of transgene expression in treated eyes relative to untreated eyes. Calculated as $2^{-\Delta\Delta CT}$
